# Correction to: A retrospective, repeated, cross-sectional study assessing changes in the prevalence of apical periodontitis associated with the endodontic status and DMFT scores

**DOI:** 10.1038/s41415-025-9387-7

**Published:** 2025-11-28

**Authors:** Chetan Morjaria, Filippo Cavalli, Francesco Mannocci

**Affiliations:** Department of Endodontics, Centre for Oral, Clinical and Translational Sciences, Faculty of Dentistry, Oral and Craniofacial Sciences, King´s College London, London, United Kingdom

Journal correction note:

*Br Dent J* 2025; DOI: 10.1038/s41415-025-8644-0

When this article was originally published, the authors were listed in the incorrect order. The correct order of the authors is Chetan Morjaria, Filippo Cavalli, and Francesco Mannocci.

The journal apologises for any inconvenience caused.

